# Best clinical practice recommendations for the management of symptomatic hemorrhoids via laser hemorrhoidoplasty: the LHP recommendations

**DOI:** 10.1007/s10151-024-03022-1

**Published:** 2024-11-23

**Authors:** The LHP Recommendation Development Group, P. C. Ambe, G. P. Martin-Martin, N. Vasas, I. Piponski, I. H. Roman, J. D. P. Hernandez, H. Ma, H.C. Lin, G. Weyand, L. Mazlan, L. J. García Flórez, K. Wolff, M. Dessily, C. Wang, V. Dobricanin, W. Yang, T. Bruketa, X.D. Zeng, S. Avdicausevic, Z.G. Zhang, S. Wais, S. Kalaskar, Z. Cui, I. B. Pestonit, Y.Q. Cao, A. Suárez Sánchez, D.L. Ren, E. Vargas Castillo, D. Zheng, B. Bogdanic, A. Wölk, Y. Yao, S. Issaad, T. Nasser, X.T. Guo, M. M. Nagar, M. Merkle, M. Ruiz-Lopez, Y. Zhang, C. Blumberg, A. A. Alam, A. C. Acosta, R. Schouten, P. Istok, C. Demtröder, Z.Y. Wang, Q. Dong, J. Wu

**Affiliations:** Witten/Herdecke University, Witten, Germany

**Keywords:** Laser surgery, Laser proctology, Hemorrhoids, Laser hemorrhoidoplasty, Minimally invasives surgery, Hemorrhoidal surgery

## Abstract

**Background:**

Laser hemorrhoidoplasty (LHP) has emerged as a novel, minimally invasive technique for managing symptomatic hemorrhoids, gaining popularity among clinicians. Despite its increasing adoption, significant variations exist in the application of LHP across different practices.

**Purpose:**

The aim of these recommendations was to spell out some basic principles and recommendations for performing a standard LHP procedure.

**Methods:**

The Recommendation Development Group (RDG) consisting of surgeons with experience in LHP were invited to formulate recommendations for the procedure. The recommendations were generated following systematic literature research and discussion amongst experts (expert opinion) where no substantial literature was available. The developed recommendations were voted upon by a panelist via the Delphi process. Consensus was a priori defined as agreement of 75% and above, with strong consensus defined as 85% and above.

**Results:**

The RDG developed 21 recommendations that were voted upon by 49 panelists. Consensus was reached for all 21 recommendations after the first Delphi round, including 16 recommendations with strong consensus.

**Conclusion:**

The RDP offers a comprehensive suite of guidelines to enhance the safety and efficacy of standard LHP procedures. Out of 21 detailed recommendations, 16 reached strong consensus, collectively addressing the full spectrum of LHP procedures—from laser settings and preoperative preparations to perioperative strategies and postoperative care. This coherent framework is anticipated not only to standardize but also to refine the LHP technique across the board, thereby elevating the management of symptomatic hemorrhoidal disease.

## Introduction

Hemorrhoids remain a prevalent condition globally, affecting an estimated 5% of the US population in 1990, with more recent studies suggesting an incidence rate of 13–16% [1–5]. Characterized by pain, itching, bleeding, and prolapse, hemorrhoids require effective management strategies [6, 7]. While many cases are treatable with conservative methods [8–10], surgical interventions continue to play a critical role [11–13]. Traditional surgical approaches include excisional techniques such as the Milligan-Morgan and Parks procedures, as well as minimally invasive options like stapled hemorrhoidopexy and hemorrhoidal artery ligation (HAL) [14–18]. Recently, laser hemorrhoidoplasty (LHP) has gained attention as a minimally invasive alternative, offering reduced pain and quicker recovery times [19, 20].

Laser-based procedures in coloproctology exploit the advantages of the minimally invasive approach and tissue preservation [1]. Thus, there has been an increase in the use of laser-based techniques to manage common conditions in this field. The technique generally known as LHP uses laser energy placed at the submucosa to shrink the piles and stimulate fibrosis with fixation of the piles onto the bowel wall [2, 3]. The advantages of this technique include less pain, early patient ambulation, early return to work, and tissue preservation [4, 5]. LHP has been investigated and compared with the standard of care (hemorrhoidectomy) and has been shown to be effective in managing hemorrhoidal conditions [6–8]. Currently, there is huge heterogeneity amongst surgeons with respect to the techniques used for LHP; thus, no single technique can be identified as the standard technique thus far [9]. This renders comparison among different users or surgical groups almost impossible. With increasing numbers of surgeons adopting this technique, there is therefore a need to set up some basic principles and recommendations on how to perform LHP. The aim of this work, therefore, was to create best clinical recommendations for LHP.

## Methods

The initiative to standardize laser treatment protocols was sparked at the PROCTOM Expert Meeting held in Malaga, Spain, in June 2023. Organized by Biolitec Biomedical Technology GmbH, Jena, Germany, a producer of laser solutions, that meeting was part of an ongoing program aimed at enhancing quality of care and providing training for surgeons performing laser interventions in coloproctology. The heterogeneity amongst users across the different interventions was seen by all (over 90) participants as problematic and the need for standardized treatment protocols was uniformly agreed upon. All participants at that meeting agreed to be contacted by email in an endeavor to discuss and establish recommendations for laser interventions in proctology. Moreover, known experts on laser surgery in coloproctology were identified from relevant publications or through Biolitec representatives. All identified surgeons were then invited by email to an introductory video conference. At that time the idea was extensively discussed. Thereafter, all interested surgeons were asked to fill out a screening questionnaire with queries about surgical training and expertise in laser-based proctologic surgery. That information was cross-checked against available publications and numbers of ordered laser fibers to confirm top users with corresponding expertise in the different interventions to serve as group leaders (steering committees) for the selected indications. A face-to-face meeting took place on September 28 2023, in Vilnius during the ESCP annual meeting.

Given the scarcity of high-quality publications, the Delphi method was selected as the optimal approach to mitigate bias from expert opinions during the recommendation formulation process. This method facilitates a more objective consensus among experts [10, 11]. It was agreed upon a priori that at least 75% agreement is necessary for consensus [12, 13]. Also, a maximum of three voting rounds was defined a priori [14, 15], each rounding lasting 14 days. Similarly, statements with at least 85% agreement would be declared strong consensus [16, 17].

A systematic literature search for available publications related to LHP was performed using the following search strategy: “Laser Hemorrhoidoplasty OR Laser HemorroidoPlasty OR LH OR LHP OR Laser hemorrhoidal surgery OR Laser hemorrhoidal procedure”. The search was limited to articles published in English language up to October 2023. Case reports, experimental studies, technical papers, conference papers, and narrative review articles were excluded. The articles generated by the search were included in an LHP library which was made available to the panelists. All participants were encouraged to send in any publications that were not part of that library.

Subsequently, panelists were invited to submit inquiries and feedback on all aspects of LHP, drawing from their review of the LHP library and their personal clinical experiences with the procedure. This step was crucial to ensure that the recommendations covered a comprehensive range of practical concerns. Equally, members of the steering committee were invited to identify and submit questions and controversial details regarding the LHP procedure based on their profound clinical experience and from currently available literature. All submitted questions and comments were scanned by members of the steering committee and used to formulate the Delphi questions (Qs).

Answers to the Qs were suggested and discussed by members of the steering committee after appraising the available literature regarding the level of evidence [18, 19]. Each Q was commented upon using available publications. Where low, very low, or no evidence was available, expert opinion was added following discussion amongst experts [19]. Discordances amongst experts were resolved by open discussions moderated by the project leader PCA. Finally, a clear response to each Q was formulated with a corresponding evidence level (strong, moderate, low, very low, or expert opinion). For statements with low and very low evidence level, expert opinion was added to reflect daily clinical practice. The completed Qs with corresponding responses (Rs) and the accompanying commentaries were discussed once more in the steering committee with minor corrections and/or rephrased before being cleared for the Delphi rounds.

The online tool Zoho CRM Plus-Survey was used for the Delphi process [20]. The Qs and their corresponding responses and commentaries were uploaded on the server and a link to the survey was generated, which was mailed to all panelists for voting. Voting stopped 14 days after initiation and the link was deactivated.

## Results

In September 2023, the initiative commenced, culminating in an inaugural video conference on the 14th of that month. An initial cohort of 90 experts in laser proctology was solicited, from which 60 specialists worldwide expressed interest. Ultimately, 48 of these experts actively contributed throughout the project, forming the core of the Recommendation Development Group (RDG). The composition and credentials of the RDG are summarized in Table 1

**Table 1:** Summary of the origins of the members of the RDG

Country	Participants
China	16
Germany	9
Spain	8
Croatia	2
Other^a^	1

^a^One surgeon from each of the following nations: Belgium, Egypt, France, Hungary, Jordan, Malaysia, Montenegro, Morocco, Serbia, Slovakia, Switzerland, the Netherlands, UK

**Fig. 1:**
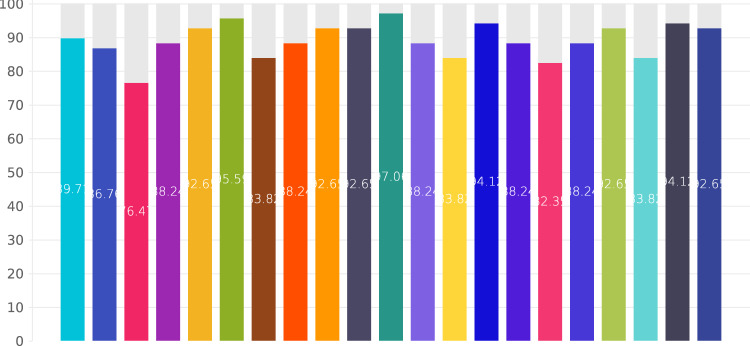
Results of the Delphi process for the 21 Qs and Rs

From the wealth of current literature and their collective expertise, the RDG formulated 21 deliberative questions (Qs) pertaining to LHP. These questions, accompanied by expert commentary, yielded 21 corresponding responses (Rs) or Delphi statements. When put to a vote among the 48 RDG panelists, all statements achieved over 75% consensus in the initial Delphi round, thereby concluding the voting process. Of these, 16 statements reached a strong consensus, defined by an agreement exceeding 85%, and the remaining five achieved consensus. The results of Delphi voting are depicted in Fig. 1.

1. **What are the main indications for LHP?**

Five randomized controlled trials (RCTs) have investigated LHP vs. excision for the treatment of symptomatic grade 2 and 3 hemorrhoids. A double-blinded RCT by Naderan et al. [21] from 2016 investigated LHP vs. hemorrhoidectomy in 60 patients with symptomatic grade 2 and 3 hemorrhoids in a 1:1 design. Another double-blinded RCT by Poskus et al. [22] from Vilnius investigated LHP vs. mucopexy vs. hemorrhoidectomy using a 1:1:1 design including 120 patients with symptomatic grade 2 and 3 hemorrhoids. Shabahang et al. [23], Lim et al. [24], and Cemil et al. [25] also included only patients with grade 2 and 3 hemorrhoids in their RCTs. Moreover, grade 2 and 3 hemorrhoids were the most common indications for LHP in published systematic reviews [26–29]. In the systematic review by Lakmal et al. [26] published in 2021 including 1937 patients from 19 studies, LHP was performed for grade 2 and 3 hemorrhoids. Similarly, grade 2 and 3 hemorrhoids represented the indications for surgery in a large majority of the studies included in the systematic review by Longchamp et al. [28]. A similar spectrum of indications was reported in the systematic reviews by Lie et al. [27] and by Wee et al. [29].

1. *Symptomatic grade 2 and 3 hemorrhoids represent the standard indications for LHP as a single intervention (without additional procedures like HAL or mucopexy).*
  *Strong consensus: 89.71%*

Q2. **Should devascularizing techniques like hemorrhoidal artery ligation (HAL) be routinely used as an adjunct to LHP?**

Although LHP alone represents a good procedure for symptomatic grade 2 and 3 hemorrhoids, it may be used in combination with other non-excisional techniques, especially mucopexy and/or devascularization of the hemorrhoidal tissues via ligation of the hemorrhoidal arteries. Hemorrhoidal dearterialization is generally performed via ligation of the hemorrhoidal artery as reported by Morinaga in 1995 [30] and aims at reducing blood flow into the hemorrhoidal plexus. Devascularization of the hemorrhoidal tissue during LHP may be performed with or without ultrasound guidance. In an RCT from Iran by Shabahang et al. the outcomes of LHP vs. hemorrhoidectomy were investigated in 80 patients in a 1:1 design. In that study, HAL and mucopexy were performed using a running 2-0 Vicryl in the LHP group [23]. In a recently published investigation from Austria, Jain et al. used ligation of the hemorrhoidal artery as well as mucopexy in addition to LHP in 30 patients with symptomatic grade 2–4 hemorrhoids [31].

Bleeding complications, e.g., postoperative hemorrhage, hematoma, and acute thrombosis, represent the most common short-term morbidities following LHP. The rate of postoperative bleeding following LHP has been reported at about 0–7.6% [32–34], while the incidence of acute thrombosis has been reported to be as high as 4–10% [35, 36]. The risk of postoperative bleeding following LHP with and without HAL was investigated in a recently published RCT by Lim et al. from Malaysia. The outcomes of 38 patients undergoing LHP alone were compared to those of 38 patients undergoing LHP followed by HAL using 2-0 Vicryl without Doppler guidance. Postoperative bleeding in this study was graded using a visual rating score where “0” corresponded to no bleeding, “1” indicated mild bleeding defined as minimal trickling or spotting, bleeding not requiring surgical intervention was graded as moderate “2”, while any bleeding with a drop in hemoglobin level and/or requiring surgical intervention was graded as severe “3” [24]. Irrespective of potential limitations regarding the rather subjective nature of the grading system used in that study, no significant difference was seen amongst both arms regarding the risk of postoperative bleeding. In contrast, the overall rate of postoperative hemorrhage (36.8% vs. 23.7%) was higher in the LHP-HAL group compared to the LHP group in that RCT. The findings from that RCT therefore suggest that there is little evidence for the routine use of HAL following LHP with the goal of reducing postoperative bleeding complications. However, the data reported in that RCT must be interpreted with caution due to possible bias in grading of postoperative hemorrhage. A crucial issue regarding the risk of postoperative bleeding complications following LHP with HAL seems to be the sequence of events. In both RCTs by Shabahang et al. and Lim et al., HAL was performed after LHP [23, 24]. A reversed sequence, starting with HAL prior to LHP, was chosen by Jain et al., who reported just one case of mild postoperative bleeding and acute thrombosis each in the LHP group with 30 patients [31]. The findings reported by Jain et al. are largely in accordance with the experience of a large majority of the experts in the RDG.

The techniques discussed above should not be confused with the Doppler-guided technique of hemorrhoidal devascularization (HeLP) using laser energy [37, 38]. Proponents of this technique see advantages in the minimally invasive means of addressing the terminal hemorrhoidal arteries without the need for sutures. Another aspect of this technique is the possibility of managing all the terminal branches of the superior hemorrhoidal artery. This procedure, however, is totally different from the standard LHP and therefore is out of the scope of this project.

R2: *HAL with or without ultrasound guidance can be performed in combination with LHP for grade 2/3 hemorrhoids, beginning with HAL prior to LHP.*
  *Strong consensus: 86.76%*

Q3. **What is the role of LHP in the management of grade 4 hemorrhoids?**

The management of grade 4 hemorrhoids with LHP has been reported in the current literature in a rather small number of cases. Patients with grade 4 hemorrhoids represented 23% of the LHP group in the study by Jain et al. [31]. Patients with grade 4 hemorrhoids constituted 38% and 46% of the study population in two prospective investigations by Mert [39] and Khan et al. [36], respectively. The available literature thus seems to support the use of LHP in selected patients with grade 4 hemorrhoids.

Because addressing the prolapse represents a relevant outcome from the patient’s perspective, the RDG recommends combining LHP with HAL and mucopexy. This technique was used in a German cohort by Weyand et al. to manage grade 4 and prolapsed hemorrhoids [40]. A short segment mucopexy was needed in 253 of the 499 patients included in their study. The findings from that study suggest that the management of grade 4 hemorrhoids using LHP in combination with mucopexy is feasible. However, there seems to be some increase in the risk of morbidity, especially in this cohort.

According to expert opinion, LHP can be used with additive techniques like HAL and/or mucopexy to manage grade 4 hemorrhoids by experienced surgeons, especially after conservative management of an acute prolapse. The patient must be informed not only about the increased risk of morbidity and recurrence but also about the increased risk of pain associated with the use of additional techniques besides LHP.

R3: *Grade 4 hemorrhoids can be managed via a combination of LHP, HAL, and/ or mucopexy based on surgeon’s expertise and patient’s expectation.*
  *Consensus: 76.47%*

Q4. **How many piles can be managed at the same time?**

While the indication for LHP is largely stated in most of the available publications, the number of piles managed was reported in very few articles. In the article by Weyand et al. two and three segments (piles) were managed in 1/3 and 2/3 of their study population, respectively [40]. Mert on the other hand reported treatment of just one quadrant in 28%, two quadrants in 28%, three quadrants in 20%, and four quadrants in 4% of her study population [39], while a median of three piles (range 1–6) were treated in the study by Faes et al. [33]. In the latter study, one case of continence disturbance was reported following LHP [33]. This rather uncommon complication after LHP may be secondary to shrinkage of many piles following extensive LHP treatment (multiple piles) in a patient with preoperatively suboptimal continence. The wide heterogeneity in the number of piles treated in published studies renders specification impossible. There is a strong consensus amongst members of the RDG to address only pathologic piles to prevent overtreatment.

R4: *Only pathologic hemorrhoids should be treated.*
  *Strong consensus: 88.24%*

Q5. **Can external hemorrhoids and skin tags be managed at the same time with LHP?**

Skin tags were removed in 28% of the German cohort by Weyand et al. [40]. Similarly, skin tags were excised in 53.3% of cases in the study by Jain et al. [31] and in 40% of cases in the Swiss study by Faes et al. [33]. While this practice appears to be in accordance with patient expectation, there is no evidence in the current literature to recommend the management of external hemorrhoids and skin tags as part of the standard LHP procedure. The RDG cautions toward the use of the laser for the shrinkage of external hemorrhoids because of a hypothetical risk of fistula formation. Moreover, additional procedures may increase the risk of complication and postoperative pain.

R5: *Management of external hemorrhoids and skin tags may be considered in individual cases (patient’s expectation) but should not represent an elementary aspect of the standard LHP procedure.*
  *Strong consensus: 92.65%*

Q6. **Can LHP be routinely combined with other proctological procedures?**

There is hardly any data in the current literature looking into the combination of LHP with other procedures. In the retrospective study by Weyand et al. four fistulectomies, seven polypectomies, and 21 fissurectomies were performed during LHP [40]. As stated above (comments to Q5), the most relevant advantage of LHP, i.e., less pain, may be jeopardized by additional procedures around the anoderm. Also, the theoretical risk of morbidity increases with additional procedures. Nonetheless, addressing other pathologies at the same time with LHP may be reasonable in some cases. The coexistence of both symptomatic piles and a chronic anal fissure in a patient presenting with pain, for example, warrants a simultaneous management of both pathologies. Similarly, polypectomy can be safely performed during LHP. The decision for additional procedures during LHP may be reached in individual cases as a shared decision between surgeon and patient after considering the patient’s expectations and surgeon’s expertise.

R6: *Additional proctological procedures can be combined with LHP in individual cases but cannot be considered as routine.*
  *Strong consensus: 95.59%*

Q7. **What are common contraindications for LHP?**

So far, contraindications for LHP have not been widely reported in the literature. However, active inflammatory processes like abscesses, proctitis, and undrained fistula may increase the risk of complication following LHP [41]. Therefore, surgery should be rescheduled in such cases. Uncontrollable hemorrhagic conditions with high risk of bleed may constitute relative contraindications.

R7: *Acute inflammation such as abscess, proctitis, and fistula represent absolute contraindications for LHP.*
  *Consensus: 83.82%*

Q8. **What is the role of LHP in managing recurrent hemorrhoids?**

The risk of recurrence may be seen as the Achilles tendon of LHP. In an RCT by Naderan et al. investigating LHP vs. hemorrhoidectomy for grade 2 and 3 symptomatic hemorrhoids, the rate of recurrence was 6.7% vs. 10% after 12 months for the LHP vs. hemorrhoidectomy, respectively [21]. In a retrospective study by Gambardella et al., the rates of recurrence following LHP were 1.3% after 6 months, 9.4% after 12 months, and 21.6% after 24 months [42]. In another retrospective study by Ram et al. from Israel 10.5% of the cohort of 162 patients undergoing LHP had undergone previous intervention for hemorrhoids [43]. In a recently published paper by Dursun et al., the risk of recurrence was 50% in 12 patients with grade 4 hemorrhoids compared to 17.6% in 91 patients with grade 2 and 3 hemorrhoids [41]. While the small number of cases undergoing LHP for grade 4 hemorrhoids in this study appears to be in line with the current literature, the high rate of recurrence for this group may be easily explained via the sample size. Moreover, it was not stated whether mucopexy was added to LHP for patients with grade 4 hemorrhoids in this study. Nonetheless, these results argue for a grade-dependent risk of recurrence. In the study be Faes et al., 49 of 50 patients treated with LHP reported that they would recommend LHP to other patients and relatives. After 5-years follow-up, 64% would still recommend LHP [33]. Because LHP is an organ-preserving procedure, the risk of recurrence would most probably remain an issue that needs to be discussed with the patient. The expert opinion is that LHP could still be a good option following recurrence.

R8: *LHP could still be a good option following recurrence and should be discussed with the patient on an individual basis.*
  *Strong consensus: 88.24%*

Q9. **What is the optimal bowel preparation prior to LHP?**

There is a high degree of heterogeneity on bowel prepping prior to LHP in the literature. An enema given prior to surgery has been reported in many studies and seems to represent the most favored method of bowel prepping in this setting [39, 44, 45]. In an RCT from Vilnius by Poskus et al., lactulose was given 1 day prior to LHP for bowel prepping [22]. So far, there is no standardized technique for bowel prepping prior to LHP. The timing of bowel prepping should be well chosen, e.g., enema 2 h prior to LHP [39, 46, 47] or the day before surgery [22]. The rationale behind this timing is to reduce the risk of spillage of feces during and immediately after LHP as a means of reducing the risk of infection of the punction sites. For patients needing colonoscopy prior to surgery, LHP can be performed after colonoscopy without further prepping. Expert opinion is to omit bowel prepping for standard LHP or use an enema 2 h prior to surgery for more extensive procedures requiring HAL or mucopexy in combination with LHP.

R9: *Bowel prepping may be omitted prior to standard LHP.*
  *If needed, a simple enema may be given about 2 h prior to surgery.*
  *Strong consensus: 92.65%*
Q10. **What is the role of perioperative antibiotic prophylaxis for LHP?**

The need for single-shot antibiotic prophylaxis specifically for LHP has not been systematically investigated. Therefore, the published data largely represents institutional standards rather than following established protocols. Despite this limitation, most published papers reporting on perioperative antibiotics used a combination of a second- or third-generation cephalosporine and metronidazole [21, 22, 24, 42]. The use of a single antibiotic for this purpose has also been reported in recent literature, e.g., Brusciano et al. used 2 g ceftriaxone i.v. [32], while the Canteralla et al. used 1.2 g of Augmentin as prophylactic antibiotic [48]. Interestingly, perioperative antibiotic prophylaxis was either not given or not reported in a huge proportion of available papers on LHP. Therefore, there is no solid data to back the routine use of prophylactic antibiotics in patients undergoing LHP. The indication for prophylactic antibiotics therefore should be made on an individual basis.

R10: *Routine single-shot antibiotics can be omitted during LHP. Antibiotic prophylaxis should be considered in individual cases on the basis of patient’s risk factors.*
  *Strong consensus: 92.65%*
Q11. **What is the optimal wavelength for LHP?**

Two wavelengths, 980 nm and 1470 nm, have been widely used to deliver the laser energy within the pile to induce shrinkage of the hemorrhoidal tissue [49–51]. High-quality evidence on the efficacy of the 980-nm wavelength is available from RCTs, mainly from the Asian Pacific region [21, 24, 35, 44]. Equally, high-quality evidence for the 1470-nm wavelength is available from published RCTs and prospective studies [22, 23, 32, 34, 36]. So far, there is no comparative study investigating both wavelengths for this indication.

R11: *Both 980 nm and 1470 nm wavelengths can be safely used for LHP.*
  *Strong consensus: 97.06%*
Q12. **What is the optimal laser setting with regards to laser power in watts for LHP?**

While the optimal wavelengths for LHP are clear, the only other undisputable technical aspect about the setting of the laser applicator is the pulse mode of energy application. Wide heterogeneity can be seen in the duration of application (pulse duration) and the amount of energy per pulse. In a retrospective investigation from Iran by Jahanshani et al., a pulse mode of 3 s duration with a power of 15 W was the reported setting using the 980-nm wavelength [52]. A similar setting was chosen by Naderan et al. in their RCT, but for the fact that the pulse duration was set at 1.2 s [21]. The same wavelength and the pulse mode with 13 W for 1.2 s was the preferred setting for Mert [39]. The wide spectrum of possible laser generator settings is further demonstrated in the report by Mohammed et al. using 5 W for 3 s in the pulse mode [53].

A similar scenario is easily identified for the 1470-nm wavelength. Gambardella et al., for example, used the pulse mode with 8 W for 3 s in their retrospective cohort [42]. This setting was used in the RCT by Poskus et al. [22] as well as in the observational study from the Netherlands by Boerhave et al. [54]. In a Turkish prospective investigation, the pulse mode was set at 10 W for 5 s [55]. Lower [56] and higher [31] performance/energy settings have been reported for the 1470-nm wavelength in the current literature (Table 2).

**Table 2:** Recommended laser settings

λ (nm)	Power (W)	Single pulse length duration (s)
980	12–15	1.2
1470	8–12	3.0

R12:
  *For laser machines employing 980 nm wavelength, a setting of 12–15 W per pulse (1.2 s) can be recommended.*
  *For laser machines employing 1470 nm wavelength, a setting of 8–12 W per pulse (3 s) can be recommended.*
  *Strong consensus: 88.24%*
Q13. **What are the anesthesiology options for patients undergoing LHP?**

LHP has been performed using various anesthesia techniques including local, regional, and general anesthesia [33, 36, 39, 53, 57]. Noori from Iraq reported a laser procedure in 150 patients with grade 3 and 4 hemorrhoids under local anesthesia using a 20-ml mixture containing 2% lidocaine with adrenaline and 0.5% bupivacaine with 5 ml sodium bicarbonate [58]. LHP was performed either under spinal or general anesthesia in the vast majority of studies [45, 51, 59, 60]. Also, saddle block has been reported as a good anesthesia option in this setting [61]. While the role of local anesthesia in this setting is not largely reflected in the current literature, clinical experience amongst experts suggests some potential for performing LHP under local anesthesia. Pre-treatment with anesthetic ointment for at least half an hour prior to surgery may facilitate the injection of local anesthesia from a patient’s perspective. The efficacy of local anesthesia may be augmented by pudendal block. Nonetheless, the need for additional sedation should always be considered and consent for anesthesia should be sorted prior to surgery.

R13: *LHP can be performed in local, regional, and general anesthesia.*
  *Consensus: 83.82%*
Q14. **What is the right technique for LHP?**

**Fig. 2:**
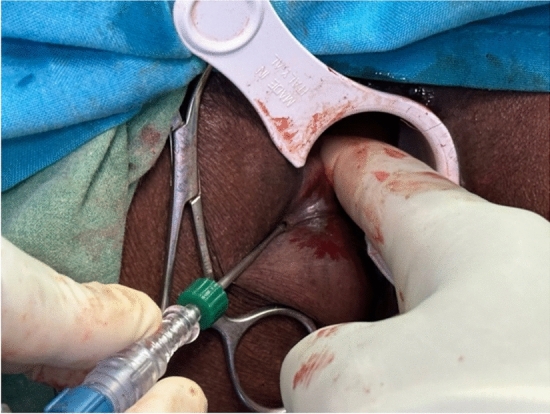
Introduction of the LHP probe into the submucosa plane with digital guidance via a palpating finger

**Fig. 3:**
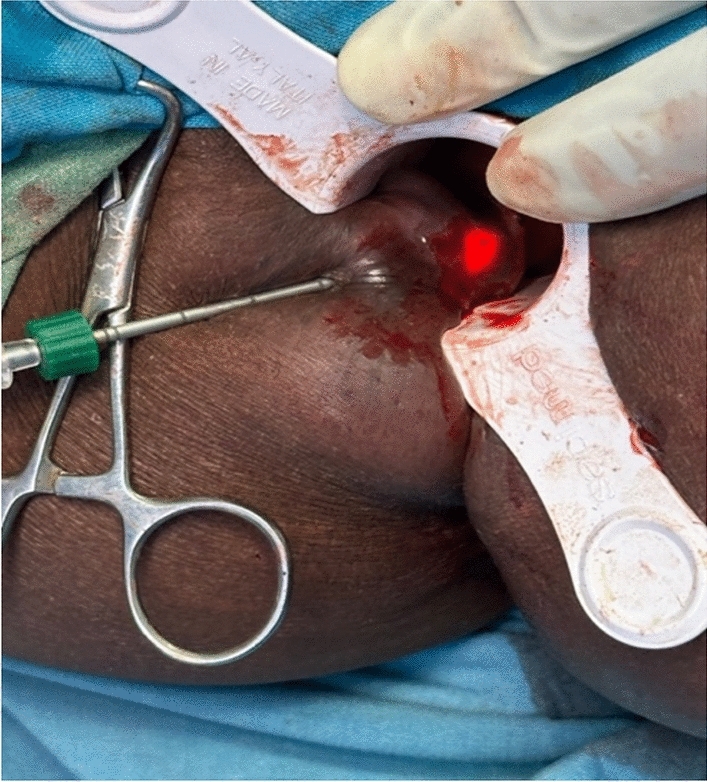
Visual control of laser probe position

The minimally invasive nature of LHP is largely based on the means of access to the hemorrhoidal tissue. The laser probe is introduced by puncturing the base of the hemorrhoid at the anal verge and advanced in the submucous plane slightly above the dentate line. For this purpose, a small incision is made using a blade, electrocautery, or the laser probe itself (Fig. 2). The laser probe is gently advanced in a controlled manner via palpation and visual control using the indicator light (Fig. 3) and the energy is applied as recommended in R15.

Direct application of the laser energy on the mucosa via a trans anal access [45] does not represent part of the standard LHP. In a study by Elsheikh et al. [62], 80 J was applied onto the feeding vessels via the anus as the first step in their treatment protocol. Equally, introducing the laser probe via an incision above [43] or at the level of the dentate line [63] is not part of the standard LHP procedure. In the latter study bleeding complications were recorded in as high as 8.8% [63]. Expert opinion is that trans anal manipulations during LHP (besides HAL and mucopexy) in the sense of direct energy application onto the mucosa as well as incising the mucosa to introduce the laser probe should be avoided because of the increased risk of morbidity.

R14: *The introduction of the laser probe is performed via a small incision at the anal verge, then the probe is gently advanced in the submucous space under digital and visual (indicator light) control.*
  *Strong consensus: 94.12%*
Q15. **What is the optimal approach and energy for a standard LHP strategy regarding the number of pulses per pile?**

The number of shots per pile has not been clearly defined in the current literature. Five shots were used by Mert per pile [39], while Mohammed et al. used 5–6 shots per pile [53]. Interesting, only three shots per pile were needed in the RCT by Naderan et al. [21]. Similarly, Alsisy et al. routinely used three shots, while three additional shots could be optionally added [35]. Higher numbers of shots (10–12) per pile were given by Brusciano et al. [32]. Besides the number of shots, the total energy per pile may represent a better parameter as this has been somehow more uniformly reported across different studies. Poskus et al. reported a maximum energy of 250 J per pile [22], while Khan et al. used 150–350 J depending on the size of the pile [36]. While there is hardly any evidence for specifying the number of shots per pile, a clear correlation has been demonstrated between the amount of energy and the risk of morbidity [40].

**Fig. 4:**
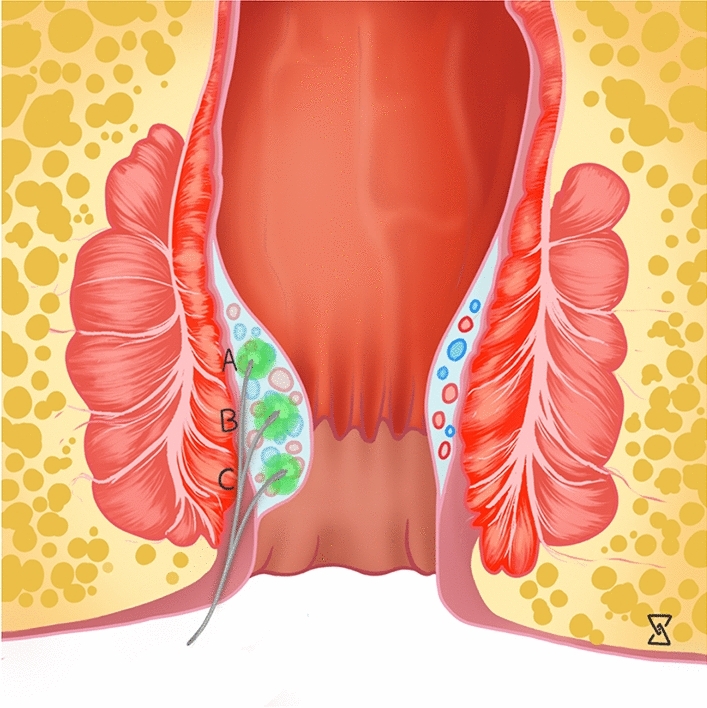
Note the different positions (A-C) of the laser probe for a standard LHP procedure

**Fig. 5:**
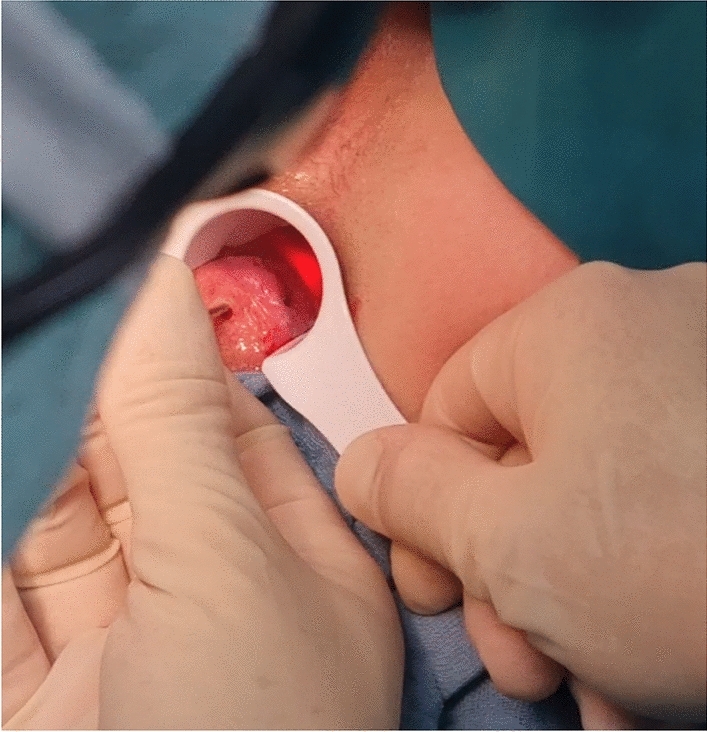
Note the indicator light above the dentate line

**Fig. 6:**
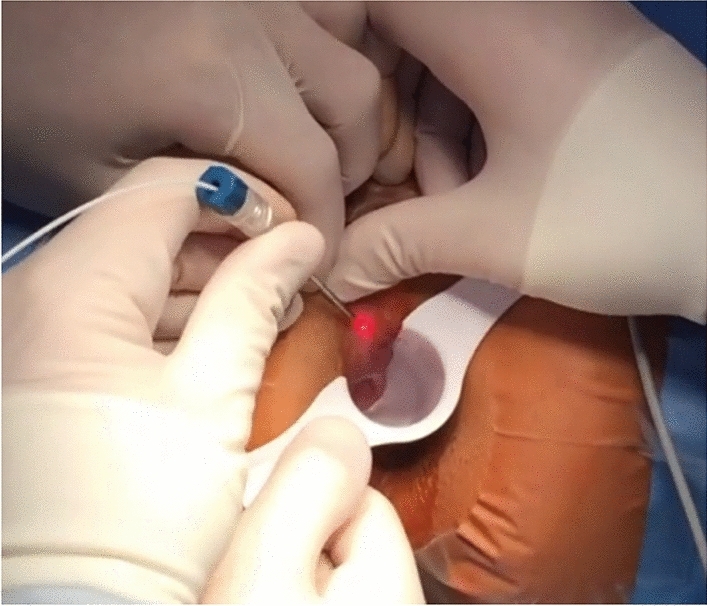
Note the final position below the dentate line
  *Additional pulses may be needed depending on the size of the piles and surgeon’s expertise. However, the maximum energy per pile should not exceed 350 J.*
  *Strong consensus: 88.24%*
Q16. **What is the role of local cooling during LHP?**

R15: *For standard LHP (grade 2/3 hemorrhoids)*
  *2 pulses about 0.5–1 cm above the dentate line *(Fig. 4)
  *3 pulses at the level of the dentate line *(Fig. 5)
  *3 pulses below the dentate line *(Fig. 6)

Cooling has been achieved via ice-cooled finger for 30–60 s [59] or cold wet gauze [31] or cold packs [23, 31, 33] or even ice packs for about 30–45 s [21, 35]. The rationale behind cooling following LHP is based on two beliefs: to reduce the effect of the energy dissipated onto the mucosa and to reduce swelling. While an ice-cooled finger or a cold wet gauze sounds reasonable for this purpose, a similar effect may be difficult to accomplish using cold packs and ice packs.

R16: *Local contact cooling measures may be performed at surgeon’s discretion in accordance with institutional standards.*
  *Consensus: 82.35%*
Q17. **What is the role of local compression following LHP**

In the study by Gambardella et al. local compression following LHP was executed with a sponge that was left in place for 12 h following surgery [42]. In another publication from the same group by Brusciano et al., a tampon was used for 12 h post LHP [32]. Jain et al. used the cooling and compressing effects of cold packs within the anal canal after LHP (which was removed at the end of surgery) [31]. Attaining better results with regards to bleeding, edema, and hematoma following LHP represents a desirable goal that may be improved via local compression. However, the potentially beneficial effects of local compression must be weighed against the potentially detrimental effects of prolonged local compression (discomfort) and the need to remove the tampon or sponge, for example.

R17: *Prolonged local compression can be routinely omitted. Absorbable sponges can be used at the surgeon’s discretion.*
  *Strong consensus: 88.24%*
Q18. **Is there any role for prolonged antibiotics after LHP?**

In the study by Mert, patients were discharged following LHP with a combination therapy including a laxative, orally administered metronidazole, and oral pain medication for 1 week [39]. The rationale behind the prolonged used of metronidazole is hard to comprehend and there is hardly any data to justify the need for antibiotics beyond the perioperative prophylactic dose. Therefore, the need for prolonged antibiotics should be made on an individual basis.

R18: *Prolonged antibiotics beyond the perioperative prophylactic dose cannot be routinely recommended. *
  *Strong consensus: 92.65%*
Q19. **What is the role of anti-inflammatory and vasculoprotective adjuncts following LHP?**

The minimally invasive LHP technique has been shown to be associated with less pain compared to excisional surgical options [21–23, 26, 28, 29]. Thus, pain following LHP can be effectively managed using oral pain killers. Most of these pain killers do have an anti-inflammatory component [64]. Therefore, there is hardly any need for more intervention in this regard.

R19: *Postoperative use of anti-inflammatory suppository, ointment, and or vasculoprotective drugs cannot be routinely recommended.*
  *Consensus: 83.85%*
Q20. **What is the optimal wound dressing following LHP?**

Wound dressing after LHP has not been uniformly reported in many published studies. One study reported using some form of external dressing at the end of the operation [53]. Another study reported that 0.2% nitrofurazone ointment was applied to the outer part of the anal canal and was dressed with gauze [65]. In standard LHP cases, wound dressing aims to collect minimal secretion from the punction site in the anal verge. This minimal spotting can be sufficiently managed with a gauze.

R20: *Extensive wound dressing can be omitted following LHP. A gauze is sufficient.*
  *Strong consensus: 94.12%*
Q21. **What is the optimal follow-up interval after LHP?**

So far, there is no recommended follow-up schedule after LHP. Therefore, an evidence-based follow-up recommendation cannot be made. Follow-up should be according to surgeon’s preference and should take local healthcare standards including availability of resources into consideration.

A follow-up calendar based on expert opinion may look like:

- Postop day 1 (following outpatient surgery): interview, inspection, no instrumentalization
- Postop day 7: Interview, inspection, digital exam
- Postop days 14 and 30: Interview, inspection, digital exam, anoscopy
- Months 6, 12, 36, and 60

R21: *Follow-up could include interview, inspection, digital exam, and anoscopy at reasonable intervals*.
*Strong consensus: 92.65%*

## Discussion

Over the preceding decade, laser hemorrhoidoplasty (LHP) has matured into a key minimally invasive approach for hemorrhoidal disease management, reflecting significant advancements in proctological procedures. The rising popularity of LHP among proctologists has been tempered by considerable variations in its application, which, before these guidelines, impeded the technique’s standardization and the ability to compare outcomes across different institutions. The RDG’s objective was to codify fundamental principles for LHP execution. This endeavor was guided by a rigorous assessment of contemporary literature, underpinning the formulation of these recommendations. In areas where literature was deficient, we sought and deliberated upon expert insights. Our ultimate goal was to craft clinically significant recommendations to direct surgeons in the application of LHP. Therefore, all relevant aspects of LHP including settings of the laser machine, preoperative, perioperative, operation technique, and postoperative aspects have been addressed in these 21 recommendations.

The formulated recommendations are largely limited by the availability of high-level evidence. This is reflected by the limited number of high-quality systematic reviews of RCTs and prospective studies on LHP [26–29, 38, 49, 66]. Therefore, our aim to provide recommendations based on published data could not be reached entirely. Whenever published data was not available, expert opinions were sought and openly discussed to reach agreement amongst the experts. The formulated recommendations were then voted upon by a panelist with experience of LHP in the Delphi process [11].

Consensus was reached on all 21 recommendations following the first voting round, with 16 recommendations scoring at least 85% agreement (strong consensus). The remaining five recommendations (R3, R7, R13, R16, and R19) although scoring above 75% agreement and fulfilling the a priori determined cutoff for consensus did not reach strong consensus. In particular, the role of LHP for the management of grade 4 hemorrhoids (R3) was critically viewed and discussed. This item reached 76.47% agreement and thus consensus. Nonetheless, this finding may be interpreted in line with a reluctance of some experts to see grade 4 hemorrhoids as an indication for LHP. This reluctance is backed by the outcome data following LHP for grade 4 hemorrhoids, thereby reflecting the clinical judgement of some surgeons involved [43].

While these recommendations may not have covered all aspects of LHP, we believe that these statements would help standardize the procedure and homogenize practice. An important aspect about these recommendations is the consideration of different policies that guide healthcare in the different geographic locations of the surgeons involved. Although not specifically interrogated, issues including the choice of anesthesia, e.g., local anesthesia in areas of low income, inpatient or outpatient surgery, follow-up, cost of treatment, etc., were considered during the RDG.

A notable limitation of these recommendations is the paucity of high-quality evidence addressing certain issues. Where literature exists, it often falls short, offering only low to very low levels of evidence. Despite efforts to mitigate bias through the Delphi process, the prominence of the steering committee’s members may have subtly swayed the responses of panelists regarding specific recommendation aspects. There is a possibility that our search strategy for publications on LHP may have missed some relevant publications. Besides, it may be disputable whether a higher threshold of agreement to reach consensus may have culminated in a different result. These limitations call for further activities in this topic, especially with the expected increase in scientific publications on LHP.

Despite the above limitations, these recommendations will hopefully standardize the LHP procedure and serve as a guide for surgeons wanting to learn and practice this surgical technique. Moreover, we hope that these recommendations may harmonize LHP and enable comparison of results amongst different users/centers. Furthermore, these recommendations may be used to guide/aid study design for future investigations in this technique.

## Conclusion

The RDP offers a comprehensive suite of guidelines to enhance the safety and efficacy of standard LHP procedures. Out of 21 detailed recommendations, 16 reached strong consensus, collectively addressing the full spectrum of LHP procedures—from laser settings and preoperative preparations to perioperative strategies and postoperative care. This coherent framework is anticipated not only to standardize but also to refine the LHP technique across the board, thereby elevating the management of symptomatic hemorrhoidal disease.

## Data Availability

No datasets were generated or analyzed during the current study.
